# Astrocytic *Dagla* Deletion Decreases Hedonic Feeding in Female Mice

**DOI:** 10.1089/can.2023.0194

**Published:** 2024-02-12

**Authors:** Este Leidmaa, Alexandra Maria Prodan, Lena-Louise Depner, Joanna Agnieszka Komorowska-Müller, Eva Carolina Beins, Britta Schuermann, Carl-Christian Kolbe, Andreas Zimmer

**Affiliations:** ^1^ Medical Faculty, Institute of Molecular Psychiatry, University of Bonn, Bonn, Germany.; ^2^ Department of Physiology, Institute of Biomedicine and Translational Medicine, University of Tartu, Tartu, Estonia.; ^3^ Medical Faculty, Institute of Human Genetics, University of Bonn, Bonn, Germany.; ^4^ Institute of Innate Immunity, University of Bonn, Bonn, Germany.

**Keywords:** endocannabinoids, 2-AG, appetite, high-fat diet, sex-specific effects, sex hormones

## Abstract

**Introduction:**

Endocannabinoids and exogenous cannabinoids are potent regulators of feeding behavior and energy metabolism. Stimulating cannabinoid receptor signaling enhances appetite, particularly for energy-dense palatable foods, and promotes energy storage. To elucidate the underlying cellular mechanisms, we investigate here the potential role of astrocytic endocannabinoid 2-arachidonoylglycerol (2-AG). Astrocytes provide metabolic support for neurons and contribute to feeding regulation but the effect of astrocytic 2-AG on feeding is unknown.

**Materials and Methods:**

We generated mice lacking the 2-AG synthesizing enzyme diacylglycerol lipase alpha (*Dagla*) in astrocytes (GLAST-Dagla KO) and investigated hedonic feeding behavior in male and female mice. Body weight and baseline water and food intake was characterized; additionally, the mice went through milk, saccharine, and sucrose preference tests in fed and fasted states. In female mice, the estrous cycle stages were identified and plasma levels of female sex hormones were measured.

**Results:**

We found that the effects of the inducible astrocytic *Dagla* deletion were sex-specific. Acute milk preference was decreased in female, but not in male mice and the effect was most evident in the estrus stage of the cycle. This prompted us to investigate sex hormone profiles, which were found to be altered in GLAST-Dagla KO females. Specifically, follicle-stimulating hormone was elevated in the estrus stage, luteinizing hormone in the proestrus, and progesterone was increased in both proestrus and estrus stages of the cycle compared with controls.

**Conclusions:**

Astrocytic *Dagla* regulates acute hedonic appetite for palatable food in females and not in males, possibly owing to a deregulated female sex hormone profile. It is plausible that endocannabinoid production by astrocytes at least partly contributes to the greater susceptibility to overeating in females. This finding may also be important for understanding the effects of exogenous cannabinoids on sex hormone profiles.

## Background

Medicinally, cannabis preparations have been used to increase appetite in humans.^1,2^ They mimic the activity of endocannabinoids, lipid ligands for cannabinoid receptors. Endocannabinoids and exogenous cannabinoids are particularly potent in increasing the intake of palatable high-fat and high-sugar foods in both mice and humans.^3–5^ Cannabinoids do this by stimulating homeostatic and reward-related areas in the brain.^6^ Hedonic eating refers to consumption of food for pleasure and not to maintain energy homeostasis.^7^ Cannabinoids increase food intake and amplify the hedonic impact of food in satiated states when the homeostatic energy requirements of the body have been fulfilled indicating their key role in hedonic appetite.^6–10^ This makes the endocannabinoid system (ECS) important for understanding the mechanisms of overeating.

Infusions of 2-arachidonoylglycerol (2-AG), the most abundant brain endocannabinoids,^11^ into different brain regions stimulate feeding behavior and specifically increase the preference for high-fat and high-sucrose food.^9,12,13^ Moreover, endogenous 2-AG is elevated in the mouse brain after conditioned eating of palatable food.^14^ Similarly, in humans, both consumption and anticipation of palatable foods increased plasma 2-AG.^7,15^ 2-AG is mainly synthesized by the enzyme diacylglycerol lipase alpha (DAGLa) in the brain.^16–19^ Mice with a constitutive deletion of *Dagla* show a slight reduction in body weight (BW) owing to reduced food intake.^19,20^ A similar phenotype was observed in mice with a deletion of the cannabinoid receptor 1 (*Cnr1*).^20,21^ The differences in food intake in *Dagla* and *Cnr1* knockout (KO) mice became more obvious when the mice were fed a palatable high-fat diet.^20^

To investigate the physiological effects of the ECS on feeding at the cellular level, mice with a conditional deletion of *Cnr1* and *Dagla* have been created.^19,22^ For example, glutamatergic *Cnr1* are responsible for the well-known orexigenic effects of cannabinoids, whereas GABAergic *Cnr1* in the ventral striatum have an anorexigenic effect.^23^ *Cnr1* signaling in the hypothalamic ventromedial nucleus, forebrain, and sympathetic neurons, on the contrary, seems to be a key determinant of endocannabinoid actions on energy balance and adiposity.^24,25^ Furthermore, astrocytic *Cnr1* modulates leptin signaling and its ability to regulate glycogen storage.^26^ Although a lot is known about the cell type–specific effects of CNR1, the cell type–specific effects of DAGLa, the enzyme that produces 2-AG to regulate appetite and hedonic eating, have not yet been identified.

DAGLa is present in neurons as well as astrocytes and microglia.^27–31^ In this study, we focus on its role in astrocytes, brain cells that have recently gained attention in the modulation of feeding and metabolism.^32^ Astrocytes provide metabolic support as well as information about the metabolic state of the body to neurons.^32–35^

Our previous study indicated that the deletion of *Dagla* from astrocytes leads to different phenotypes in males and females,^31^ therefore, we pay particular attention to the aspect of sex. Sex is important when studying feeding regulation and particularly hedonic appetite, as women are more prone to weight gain and obesity than men, as well as eating for pleasure, binge eating, and other eating disorders.^36–38^ Some of the known sex differences in feeding have been attributed to sex hormones, particularly to the effects of estrogens.^39^ Human and animal studies indicate that estradiol typically decreases food intake and BW^40–43^ but increases energy expenditure and physical activity.^44–46^ Less information is available on progesterone, but it seems to oppose the effects of estrogen and increase appetite.^47,48^ Males and females also differ in their response to cannabis according to surveys, laboratory-based studies, and studies in natural settings.^49,50^ Furthermore, these differences are modulated by sex hormones.^49,51^

In this study, we investigated whether astrocytic *Dagla* deletion regulates hedonic appetite. We tested tamoxifen-inducible male and female GLAST-Dagla KO mice and their littermate controls in a set of preference tests with palatable solutions. Furthermore, we investigated whether different estrous cycle stages affect hedonic eating in females and if they interact with the effects of astrocytic *Dagla*. The sex hormone profile in female GLAST-Dagla KO and control mice was also characterized.

## Materials and Methods

### Animals

Animals with a C57BL/6J genetic background were bred and housed in standard cages in climate-controlled rooms. Mice were housed with an inverted 12 h dark/light cycle (light on 21:00 h, ZT1) and tested in the dark phase. Mice received food (standard maintenance chow V1534-300, Ssniff Spezialdiäten GmbH, Germany) and water *ad libitum*, except during experiments containing a fasting period. Tamoxifen-inducible Cre/loxP system was used to generate the mouse mutants to have *Dagla* deletion in astrocytes in adulthood and avoid developmental effects. Astrocytic *Dagla* KO line (GLAST-Dagla KO) was obtained by crossing mice that were heterozygous for the tamoxifen-inducible form of Cre (CreERT2) in the locus of the astrocyte-specific glutamate aspartate transporter (GLAST) [Slc1a3tm1(cre/ERT2)Mgoe]^52^ with homozygous *Dagla* “floxed” mice (Dagla fl/fl) mice [B6.Dagla tm1Zim].^19^

Both Dagla fl/fl controls and GLAST-Cre positive Dagla fl/fl were intraperitoneally injected with 1 mg tamoxifen (T-5648; Sigma, Schnelldorf, Germany) dissolved in 50 μL corn oil (90%) and ethanol (10%) twice daily for 5 consecutive days.^52^ Deletion of *Dagla* specifically from astrocytes and not from neurons was verified.^31^ All experiments were approved by the North Rhine-Westphalia State Environment Agency (LANUV, Landesamt für Natur, Umwelt und Verbraucherschutz) and were performed in accordance with the relevant guidelines and regulations.

For detailed information on tamoxifen injections and quantitative real-time polymerase chain reaction, see Supplementary Data.

### Food preference experiments

The mice were single-housed in standard plastic cages 2 weeks before the behavioral experiments. Measurements and experiments were carried out in the active phase of the circadian cycle (dark phase, experiment starting at ZT14). BWs were measured before tamoxifen injections and before and after each preference test. Baseline food intake was measured during 2 weeks before the start of hedonic feeding experiments. Baseline water intake was measured and mice were habituated to two test bottles with water for 4 days before preference tests. For each preference test, mice were given access to both water and a palatable solution of choice for 24 h. Standard chow pellets containing 3.89 kcal/g (9% fat, 58% carbohydrate, 33% protein) were present *ad libitum* throughout the tests. The solutions of choice were milk (5% fat), saccharin (0.1%), or sucrose (1%). For detailed information on food preference experiments, see Supplementary Data.

Male mice weighed more than females in all experiments. Thus, to show comparable results, the raw values of food and liquid intake were normalized to the BWs.

### Estrous cycle

Estrous cycle of female mice was assessed by vaginal lavages with 50 μL of sterile water and a subsequent crystal violet staining of vaginal smears.^53^ Lavages were taken at the 3 h time-point of the preference tests (ZT17, mid-dark phase). For cytological assessment, 0.1% crystal violet staining solution was prepared by mixing crystal violet powder with double distilled water. The dried slides with vaginal smears were immersed in the 0.1% crystal violet solution for 1 min and washed two times 1 min in double distilled water. Brightfield microscopy was used to visualize and estimate the cycle stages.^53,54^

### Measurements of female sex hormones

Female GLAST-Dagla KO and control mice from two separate cohorts were decapitated in the dark phase (ZT16-17). Blood samples were collected in ethylenediaminetetraacetic acid–coated tubes and centrifuged at 2000 *g* for 10 min at 4°C. The obtained plasma was transferred to new tubes and stored at −80°C. Levels of follicle-stimulating hormone (FSH), luteinizing hormone (LH), prolactin, 17-beta-estradiol, and progesterone were measured using the multiplexed hormone detection assays using LUMINEX technology (MPTMAG-49 K, RMNPMAG-83 K, and MSHMAG-21 K by Merck/Millipore). Assays were performed on a Luminex MagPix device. The analysis excluded two statistically significant outliers from progesterone measurements that were more than 10-fold higher than the group average.

Results from two cohorts of mice were pooled to have a sufficient number of animals in each estrous cycle stage. To be able to compare sex hormone levels from different cohorts and assays, the data were normalized to the average of the control group in met- and diestrus stages (100%), as those stages showed the lowest levels of the sex hormones and thus served as a baseline.

### Data analysis

Data were analyzed and plotted with GraphPad Prism, Version 10.1.0. The Shapiro–Wilk test was used to estimate whether the data were normally distributed. Statistical significance was assessed using Student's *t*-test, Mann–Whitney test for nonparametric data, one-way analysis of variance (ANOVA) with Tukey's multiple comparison tests, Kruskal–Wallis test for nonparametric data, two-way ANOVA with Sidak's multiple comparison tests, or one-sample *t*-tests/Wilcoxon tests. Statistical tests and results are given in the figure legends. Data are presented as mean±standard error of the mean.

## Results

### GLAST-Dagla KO mice have normal baseline feeding and BW

**FIG. 1.**
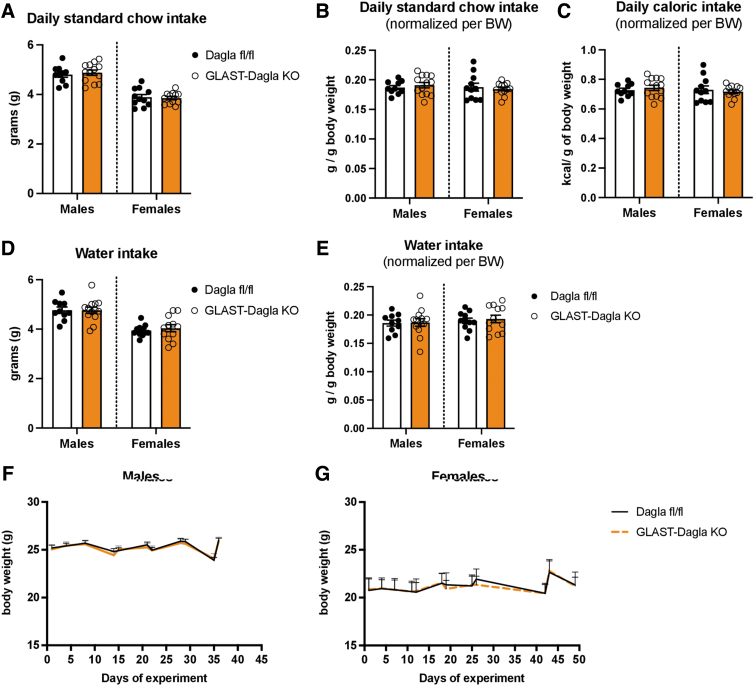
Baseline feeding and the BW are not altered in GLAST-Dagla KO mice. Standard chow intake in grams was not different between in genotypes neither in males (*p*=0.636) nor in females (*p*=0.779) **(A)**. There was also no difference when food intake in grams was normalized to BW (males: *p*=0.506, females: *p*=0.646) **(B)** nor when caloric intake was normalized to BW (males: *p*=0.51, females: *p*=0.648) **(C)**. Water intake in grams was not different between genotypes neither in males (*p*=0.969) nor in females (*p*=0.559) **(D)**. There was also no difference when water intake in grams was normalized to BW (males: *p*=0.906, females: *p*=0.692). **(E)**. Body weight did not differ in male [genotype F (1, 21)=0.042, *p*=0.84; time F (4.242, 89.08)=39.52, *p*<0.0001; time×genotype F (10, 210)=0.874, *p*=0.559]; **(F)** or female [genotype F (1, 21)=0.029, *p*=0.867; time F (2.086, 43.06)=19.47, *p*<0.0001; time×genotype F (11, 227)=0.6859, *p*=0.751]; **(G)** Two-way ANOVA followed by Sidak's multiple comparison test, *n*=10–13 animals/group. Values represent mean±SEM. ANOVA, analysis of variance; BW, body weight; SEM, standard error of the mean.

The deletion of astrocytic *Dagla* did not affect baseline intake of standard chow compared with controls (Dagla fl/fl littermates) (Fig. 1A). Whereas males, independent of genotype, consumed more standard chow than females (Fig. 1A), normalizing the intake to their BW revealed that both sexes had comparable food intake (gram per gram of BW in Fig. 1B and calories per gram of BW in Fig. 1C). A similar effect was observed for water intake, as GLAST-Dagla KO showed no differences from control mice (Fig. 1D). Again, males drank more than females but this difference disappeared after normalizing the intake to the BW (Fig. 1E). There were also no significant BW differences between the GLAST-Dagla KO and control mice of either sex (Fig. 1F, G).

### Female GLAST-Dagla KO mice have altered preference for palatable food

**FIG. 2.**
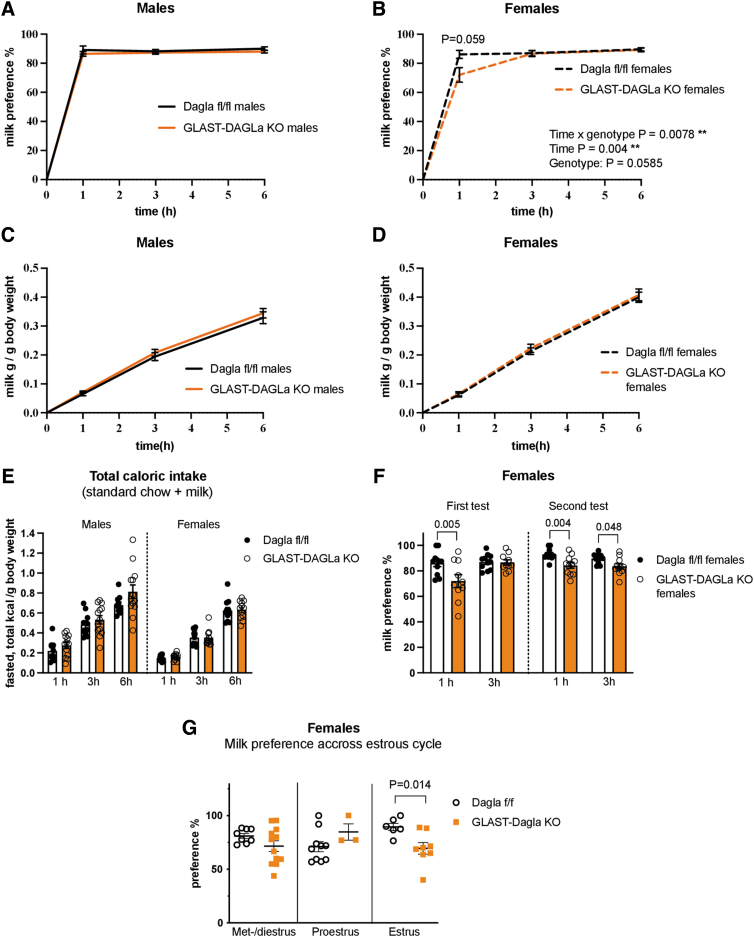
Milk preference is reduced in GLAST-Dagla KO females but not males. **(A)** Cumulative milk (5%) preference over the total amount of liquid consumed was not changed in male GLAST-Dagla KO mice compared with WT littermate controls [genotype F (1, 21)=1.305, *p*=0.266; time F (1.068, 22.44)=0.635, *p*=0.444; time×genotype F (2, 42)=0.216, *p*=0.807] **(A)**, but milk preference was reduced in female GLAST-Dagla KO mice compared with controls with a significant time and genotype interaction [time×genotype F (2, 38)=5.533, *p*=0.008; genotype F (1, 19)=4.052, *p*=0.0585; time F (1.079, 20.50)=10.1, *p*=0.004] **(B)**. Genotype did not affect milk intake neither in male [genotype F (1, 21)=0.5321 *p*=0.474; time F (1.307, 27.44)=524.1, *p*<0.0001; time×genotype F (2, 42)=0.154, *p*=0.858] **(C)** nor in female mice [genotype F (1, 20)=0.1932, *p*=0.6650; time F (1.441, 28.82)=633.8, *p*<0.0001**;** time×genotype F (2, 40)=0.054, *p*=0.948] **(D)**. Total caloric intake in the milk preference test was also not influenced by genotype in neither males [genotype F (1, 21)=0.824, *p*=0.376; time F (1.688, 35.44)=410.7, *p*<0.0001; time×genotype F (2, 42)=0.064, *p*=0.938] nor in females [genotype F (1, 20)=0.007, *p*=0.935; time F (1.450, 29.00)=362.9, *p*<0.0001; time×genotype F (2, 40)=0.353, *p*=0.705] **(E)**. Milk preference was reduced in female GLAST-Dagla KO mice compared with controls in the first hour of the test in two subsequent milk preference tests conducted a week apart. First test: genotype F (1, 19)=5.201; *p*=0.0343, time F (1, 19)=6.537, *p*=0.0193; interaction F (1, 19)=5.217, *p*=0.034. Second test: genotype F (1, 19)=8.986, *p*=0.007; time F (1, 19)=6.019, *p*=0.024; interaction F (1, 19)=2.493, *p*=0.131. **(F)** Milk preference significantly reduced the estrus stage of the cycle in female GLAST-Dagla KO compared with control mice at 1 h time-point, two cohorts of mice were pooled; met-, diestrus *p*=0.158, proestrus *p*=0.084, significant differences in estrus *p*=0.014 **(G).** Two-way ANOVA followed by Sidak's multiple comparison test, *n*=10–13 animals/group, *p<0.05 *post hoc* test **(A–F)**. Mann–Whitney *U* or Student's *t*-tests (*n*=3–12/subgroup) **(G)**. Data points represent single mice, values represent mean±SEM.

To test if *Dagla* deletion from astrocytes affects hedonic feeding, mice were given access to a 5% milk solution in addition to their normal food and water. Male GLAST-Dagla KO mice showed no differences in the preference for palatable milk solution over water, intake of milk, or their total caloric intake compared with controls (Fig. 2A, C, E). However, female GLAST-Dagla KO mice showed lower milk preference in the first hour of the test than controls (Fig. 2B). No differences were found when milk intake was measured in grams or when total caloric intake including standard chow consumption was calculated (Fig. 2D, E). A second milk preference test in the same mice confirmed these findings; again, a decreased milk preference was observed in GLAST-Dagla KO female mice (Fig. 2F). As for the first test, GLAST-Dagla KO females had a lower milk preference during the first hour of the second test (Fig. 2F).

As milk preference was only decreased in female and not male mice, we investigated a possible contribution of the estrous cycle to milk preference. As given in Figure 2G, the estrous cycle seems to modulate the phenotype as females with an astrocytic *Dagla* deletion show a significantly reduced milk preference only in the estrus stage. Moreover, when results from control females across estrous cycle were analyzed separately (using a one-way ANOVA), controls exhibited a different dynamic for milk preference compared with the GLAST-Dagla KO mice. Control mice showed a significant increase in preference for palatable milk solution in the estrus stage of the cycle when compared with the proestrus [*F*(2,21)=5.31, *p*=0.0136, Tukey *post hoc p*=0.0115], whereas this increase was absent in GLAST-Dagla KO females (Kruskal–Wallis statistic=1.659, *p*=0.436; Fig. 2G).

### GLAST-Dagla KO mice do not have altered preference for palatable food after fasting

**FIG. 3.**
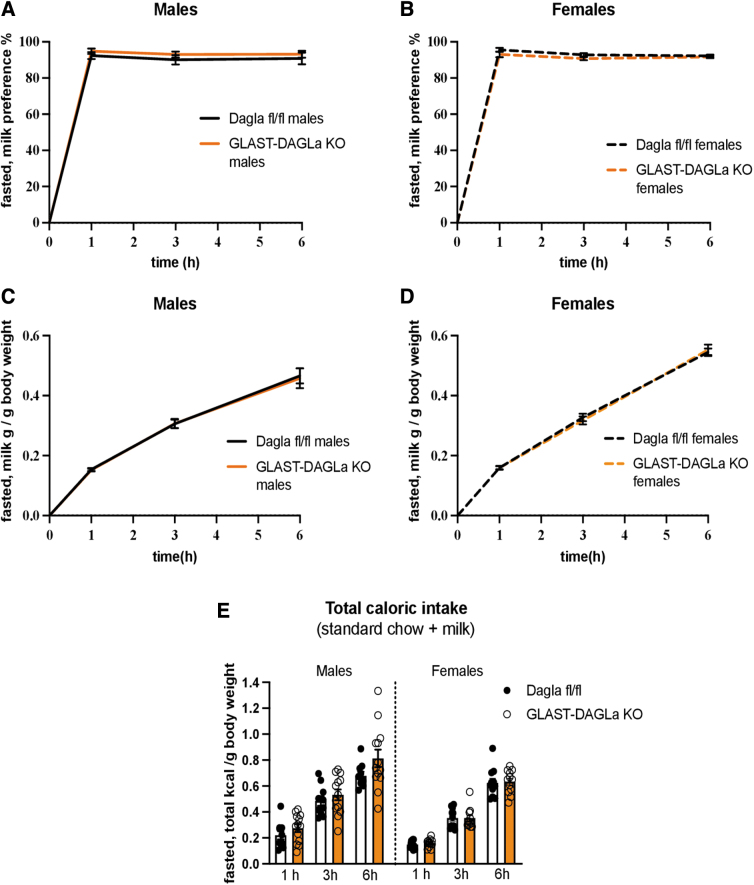
GLAST-Dagla KO does not affect milk preference after fasting. There were no genotype differences between GLAST-Dagla KO and control mice of either sex in milk preference test after fasting **(A–D)**. Cumulative milk (5%) preference over the total amount of liquid consumed was not changed in either males [genotype F (1, 21)=0.965, *p*=0.337; time F (1.076, 22.6)=1.283, *p*=0.273; time×genotype F (2, 42)=0.027, *p*=0.973] **(A)** or females [genotype F (1, 21)=2.162, *p*=0.156; time F (1.362, 28.6)=6.613, *p*=0.0095; time×genotype F (2, 42)=0.807, *p*=0.453] **(B)**. Cumulative milk intake/gram of body weight does also not differ in males [genotype F (1, 21)=0.0163, *p*=0.9; time F (1.075, 22.6)=216.2, *p*<0.0001; time×genotype F (2, 42)=0.0508, *p*=0.951] **(C)** or females [genotype F (1, 21)=0.00186, *p*=0.966; time F (1.428, 29.99)=1459, *p*<0.0001; time×genotype F (2, 42)=0.812, *p*=0.451] **(D)**. Total caloric intake (milk plus standard chow)/gram of body weight is not changed between the genotypes in male [genotype F (1, 21)=1.922, *p*=0.18; time F (1.167, 24.5)=209, *p*<0.0001; time×genotype F (2, 42)=1.862, *p*=0.168] or female mice [genotype F (1, 21)=0.0627, *p*=0.805 time F (1.78, 27.4)=482.6, *p*<0.0001; time×genotype F (2, 42)=0.0582, *p*=0.944] after fasting **(E)**. Results of two-way ANOVA (*n*=10–13/group). Values represent mean±SEM.

CNR1 antagonists reduce fasting-induced hyperphagia and mice with a *Cnr1* deletion also show reduced food intake after fasting.^23,55^ Thus, we tested how the metabolic challenge of fasting affects GLAST-Dagla KO mice. After 24 h of fasting, mice were given free access to milk, water, and chow. Neither male nor female mice showed genotype differences in milk preference, intake, or total caloric intake at any of the time points measured (Fig. 3A–D). The genotype differences that were observed in satiated females were thus abolished after fasting.

### GLAST-Dagla KO mice do not show an altered preference for either saccharine or sucrose

**FIG. 4.**
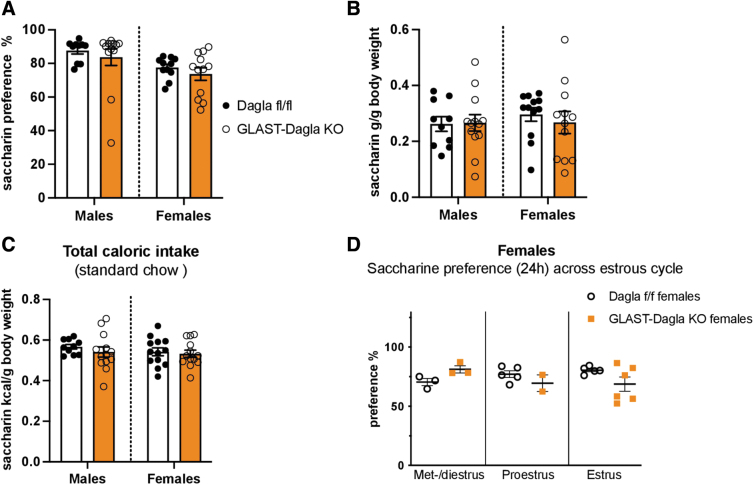
GLAST-Dagla KO mice show no differences from WT controls in the preference for noncaloric sweet-tasting saccharine solution. There were no genotype differences between GLAST-Dagla KO and control mice of either sex in the saccharine preference test. **(A)** Cumulative saccharine (0.1%) preference over the total amount of liquid consumed did not differ between GLAST-Dagla KO and control mice in either male (*U*=59, *p*=0.726) or female group (*t*(21)=0.874, *p*=0.392). **(B)** Cumulative saccharine solution intake per gram BW was not changed between the groups; either males (*t*(21)=0.095, *p*=0.925) or females (*U*=53, *p*=0.291). **(C)** Total caloric intake (from sucrose solution plus standard chow)/gram of BW was also not different between the genotypes: males (*t*(21)=0.095, *p*=0.925) or females (*U*=53, *p*=0.291). **(D)** Saccharine preference was not changed in different estrous cycle stages between female GLAST-Dagla KO and control mice at 24 h; met-/diestrus: *p*=0.1, proestrus: *p*=0.571, estrus stage: *p*=0.116. Mann–Whitney *U* or Student's *t*-tests (*n*=10–15/group, **A–C**). Student's *t*-tests (*n*=1–7/subgroup, **D**). Values represent mean±SEM.

Next, we wanted to determine if the decreased milk preference of GLAST-Dagla KO mice is because of their decreased ability to perceive sweet taste. Thus, we used the saccharine preference test as saccharine tastes sweet but contains no calories. GLAST-Dagla KO mice of either sex did not differ from controls in the saccharin preference (Fig. 4A), saccharine intake normalized to BW (Fig. 4B), or the total calories of standard chow ingested during the preference test (Fig. 4C). Differently from the milk preference test, estrous cycle did not seem to contribute to the saccharine preference in either of the genotypes (Fig. 4D). As a similar amount of saccharine solution was consumed within 24 h as milk in 1 h, the effects of estrous cycle on the 24 h time-point are shown.

**FIG. 5.**
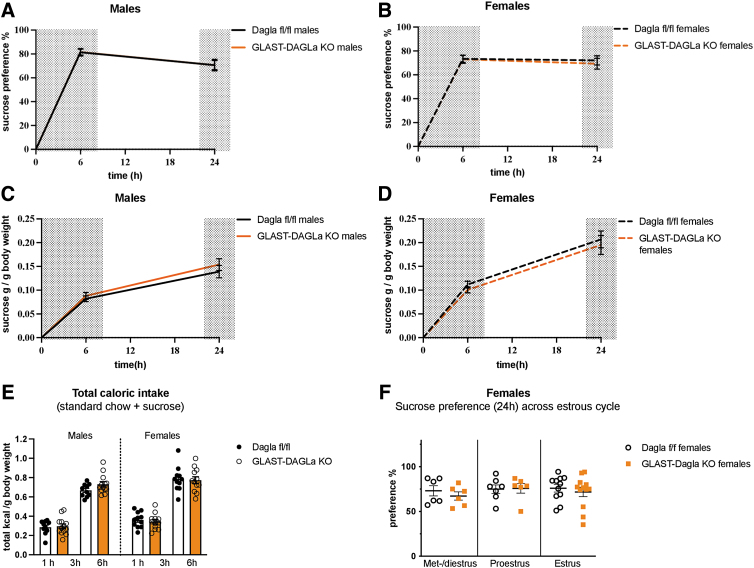
There were no genotype differences between GLAST-Dagla KO and control mice of either sex in sucrose preference test. **(A, B)** Cumulative sucrose (1%) preference over the total amount of liquid consumed did not differ between GLAST-Dagla KO and control mice in either males [genotype F (1, 21)=0.000294, *p*=0.987; time F (1, 21)=31.4, *p*<0.0001; time×genotype F (1.21)=0.00368, *p*=0.952] or females [genotype F (1, 21)=0.0982, *p*=0.757; time F (1, 21)=1.598, *p*<0.22; time×genotype F (1.21)=0.372, *p*=0.549]. **(C, D)** Cumulative sucrose solution intake per gram of body weight was not different in males [genotype F (1, 19)=0.587, *p*=0.453; time F (1, 19)=109.9, *p*<0.0001; time×genotype F (1.19)=0.568, *p*=0.46] or females [genotype F (1, 20)=0.438, *p*=0.515; time F (1, 20)=94.4, *p*<0.0001; time×genotype F (1.20)=0.000569, *p*=0.981). **(E)** Total caloric intake (from sucrose solution plus standard chow)/gram of BW does also not differ in males [genotype F (1, 19)=0.587, *p*=0.453; time F (1, 19)=109.9, *p*<0.0001; time×genotype F (1.19)=0.568, *p*=0.46] or females [genotype F (1, 20)=0.438, *p*=0.515; time F (1, 20)=94.4, *p*<0.0001; time×genotype F (1.20)=0.000569, *p*=0.981] in the sucrose preference test. **(F)** Sucrose preference does not change between different estrous cycle stages in female GLAST-Dagla KO and control mice, 24 h time-point is shown, two cohorts of mice were pooled; met-/diestrus: *p*=0.450, proestrus: *p*=0.703, estrus: *p*=0.552. Results of two-way ANOVA (*n*=10–13/group) **(A–C)**. Mann–Whitney *U* or Student's *t*-tests (*n*=6–12/subgroup) **(D)**. Values represent mean±SEM.

Next, we analyzed differences in the preference for a low percentage sucrose solution (1%), which differently from saccharine, contains calories and is more palatable than saccharine, but is less caloric and palatable than milk. As was the case for the saccharin preference, GLAST-Dagla KO did not affect sucrose preference, intake, or total caloric intake in either sex (Fig. 5A–E). The estrous cycle did also not significantly contribute to the sucrose preference in either of the genotypes (Fig. 5F). As a similar amount of sucrose solution was consumed within 24 h as milk in 1 h, the effects of estrous cycle on the 24 h time-point are shown.

### Hormonal changes in GLAST-Dagla KO females

**FIG. 6.**
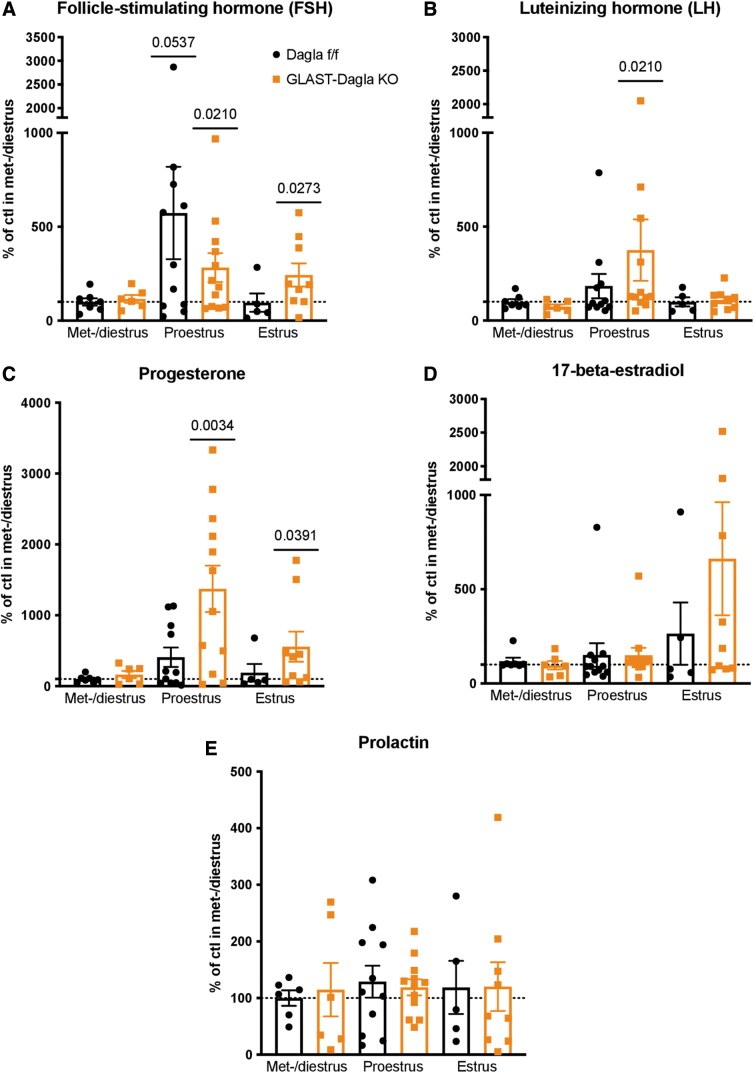
Female sex hormone profile in GLAST-Dagla KO females is altered compared with controls. The measurements were pooled from two different cohorts to obtain enough animals per estrous cycle stage and normalized to the control values (Dagla fl/fl) in met-/diestrus stage of the respective cohort (100%). **(A)** FSH was elevated in proestrus stage compared with the baseline in both GLAST-Dagla KO and control mice but in GLAST-Dagla KO mice it was also significantly increased in the estrus stage. **(B)** Luteinizing hormone was elevated in the proestrus stage only in GLAST-Dagla KO and not control mice. **(C)** Progesterone was also increased in both proestrus and estrus stages only in GLAST-Dagla KO mice. **(D)** 17-beta-estradiol was not significantly changed across the cycle but was tendentially elevated in GLAST-Dagla KO females in estrus stage (*p*=0.09). **(E)** Prolactin was not different from the baseline values in any of the estrous cycle stages. One-sample *t*-test, values represent mean±SEM. FSH, follicle-stimulating hormone.

Because of the sexually dimorphic phenotype of GLAST-Dagla KO mice observed in the milk preference test, as well as previously reported findings,^31^ we measured female sex hormones during the estrous cycle in GLAST-Dagla KO and control mice. FSH was significantly increased compared with the baseline (levels of Dagla fl/fl control group in met- and diestrus) in both genotypes in the proestrus stage. However, in the estrus stage, FSH was only elevated compared with the baseline in GLAST-Dagla KO females and not the controls (Fig. 6A). LH was also significantly increased in the proestrus stage compared with baseline levels of the control group only in GLAST-Dagla KO and not control mice (Fig. 6B).

Correspondingly, also progesterone was increased in proestrus and estrus compared with the met- and diestrus baseline levels only in GLAST-Dagla KO and not controls (Fig. 6C). 17-beta-estradiol was not significantly altered in either genotype but showed a tendency toward an increase in GLAST-Dagla KO (*p*=0.09; Fig. 6D). Prolactin was not significantly changed compared with the baseline in either genotype or throughout the estrous cycle (Fig. 6E).

## Discussion

In this study, we show that astrocytic *Dagla* deletion (GLAST-Dagla KO) reduced acute milk preference in female, but not male mice. This effect was most prominent in the estrus stage, indicating a link between astrocytic endocannabinoid production, the estrous cycle and hedonic feeding. Indeed, we found that the sex hormone profile was also altered in females with the astrocytic *Dagla* deletion, with significant changes in FSH, LH, and progesterone levels.

Endocannabinoids and exogenous cannabinoids are known for their appetite-inducing properties in rodents and humans.^3,4,56^ Recent evidence suggests that the effects of CNR1 on feeding are cell-type specific.^6^ Cell type–specific effects of *Dagla* are, however, much less studied until now. Using inducible GLAST-Dagla KO mice, we now found that astrocytic *Dagla* deletion reduced acute milk preference. However, this effect was sex-specific and seen in female but not male mice. Our observation was validated in a second test and the results align with the general appetite-inducing effect of cannabinoids. Astrocytic deletion of *Dagla* did not affect the intake of standard chow and decreased acute appetite for hedonic milk only when the animals were satiated and not fasted, suggesting that the effects were specific for hedonic and not homeostatic feeding regulation.

Female GLAST-Dagla KO showed a reduced milk preference only in the first hour of the test. This acute effect is fitting to the dynamics of the effect of CNR1 signaling on hedonic appetite.^23^ Infusions of 2-AG into the nucleus accumbens shell also increase food intake during the first hour after injection.^9^ Pharmacological inhibition of 2-AG biosynthesis, on the contrary, reduces both acute (30 min and 1 h) and short-term (14 h) high-fat diet intake.^57^ The initial approach to palatable foods during the first hour of exposure is particularly important for developing food preferences. Homeostatic food intake control mechanisms, like behavioral response to leptin, can be overridden by hedonic motivation during the first hour of novel palatable food availability.^58^ This, in turn, can affect the development of conditioned responses toward specific foods and shape eating behavior in the future.

Differently from milk preference, the genotype did not affect preference for sucrose and saccharine solutions in our study. The nutrient composition of milk is similar to foods available in natural settings in which different nutrient classes are present in one meal and seem to potentiate the rewarding value of the food^59^ as well as its postprandial effects. The higher caloric value of milk and the combination of fat and sugar make it more rewarding than 1% sucrose solution or 0.01% saccharine solution. The data from our current experiment support this. A similar amount of milk was consumed in 1 h as sucrose and saccharine solutions within 24 h. The greater hedonic value of milk might explain why we see genotype effects in milk preference but not in the tests with sweet solutions.

Previous studies have also shown that antagonists of CNR1 decrease short-term intake of food high in both sugar and fat but not food containing only sugar.^60^ This fits our results and indicates that the ECS regulates the appetite for highly palatable foods containing various nutrients. Of note, when the sucrose preference test was extended to 48 h, female but not male astrocytic *Dagla* KO mice also showed reduced sucrose preference.^31^

As only female astrocytic *Dagla* KO mice showed altered milk preference, we hypothesized that this phenotype could be affected by hormonal changes. Thus, we next looked at milk preference in females at different stages of the estrous cycle. Each phase (diestrus, proestrus, estrus, and metestrus) has its own ovarian hormone profile, with estrogen peaking during the first half and progesterone rapidly peaking during the second half of proestrus stage after which both hormones drop in the estrus stage.^61,62^ We found that the effect of astrocytic *Dagla* KO was dependent on the stage of the estrous cycle. Specifically, astrocytic deletion of *Dagla* reduced milk preference significantly in the estrus stage. In controls, but not in astrocytic *Dagla* KO mice, hedonic appetite was decreased in proestrus compared with the estrus phase. This decrease in controls fits well with the generally accepted anorexigenic effect of high beta-estradiol levels in proestrus.^40,47,63^

Therefore, we subsequently measured the levels of sex hormones in different phases of the estrous cycle to determine if genotype-specific variations could explain the reductions in appetite seen in the estrus phase in mice with astrocytic *Dagla* deletion. The sex hormone profile was indeed deregulated in GLAST-Dagla KO females. In addition to the FSH peak normally present in proestrus, GLAST-Dagla KO mice also showed increased FSH levels in the estrus stage. There was also a tendency for increased FSH levels in our previous study carried out in GLAST-Dagla KO mice^31^; this was now validated with precise discrimination between estrous cycle stages and using a larger sample size. In addition, the levels of the second gonadotropic hormone, LH, were found to be higher in astrocytic *Dagla* KO mice compared with controls in the proestrus stage.

The exogenous cannabinoid tetrahydrocannabinol has been shown to inhibit the hypothalamic–pituitary–ovarian (HPO) axis, thus leading to decreased levels of female hormones in cannabis users and deregulation of the ovarian cycle.^64–67^ In females, chronic exposure to cannabinoids also delayed sexual maturation, caused menstrual cycle disruption, depressed ovarian follicular maturation, and reduced serum concentrations of LH and sex hormones.^68^ This implies that the ECS is involved in controlling the HPO axis, possibly through negative feedback inhibition, the main function of endocannabinoids in neural tissue.^69^ It is plausible that by deleting astrocytic *Dagla*, the efficacy of this “brake” was dampened and the HPO axis became overactive. GLAST-Dagla KO mice showed reduced levels of endocannabinoids in the brain.^31^ These mice might thus have less of the natural inhibition exerted on gonadotrophin release via inhibitory G protein–coupled CNR1 stimulation.

Moreover, astrocytes, cells in which *Dagla* was deleted in our GLAST-Dagla KO mice, have been shown to regulate ovulation.^62,70^ Estradiol binds membrane-associated estrogen receptor alpha and induces an increase in free cytosolic calcium concentration astrocytes.^62,70^ Since the increase of cytosolic calcium induces the synthesis of 2-AG^71,72^ and 2-AG normally leads to negative feedback, then it is plausible that astrocytic 2-AG controls HPO axis through this mode of action. Thus, our findings further support the hypothesis that exogenous cannabinoids,^64^ as well as 2-AG produced by astrocytes, could regulate the HPO axis centrally in the hypothalamus. However, it should be noted that the deletion of astrocytic *Dagla* in our mouse line lacked region specificity, so to prove this hypothesis *Dagla* would have to be specifically deleted in the brain nuclei that regulate the HPO axis.

We found progesterone levels to be more than two-fold higher in GLAST-Dagla KO females compared with controls in both proestrus and estrus stages. Because FSH stimulates the ovaries to produce estradiol and progesterone, it is likely that elevated progesterone levels in GLAST-Dagla KO are the result of increased FSH and the deletion of astrocytic *Dagla* leads to a dysregulation of the entire sex hormone profile. Beta-estradiol levels, however, did not show any significant changes. As mentioned previously, in the estrus stage, at the first half of the dark phase in which our experiments were conducted, progesterone levels rapidly drop compared with their levels in proestrus in rodents.^61,62^ Contrary to estradiol, progesterone seems to increase appetite, probably by opposing the effects of estrogens.^43,47^ The elevated progesterone levels in proestrus in GLAST-Dagla KO mice could lead to a deregulated feeding profile across the estrous cycle.

The lack of appetite reduction in the proestrus phase in KOs compared with control mice and the subsequent rapid drop of progesterone in the estrus phase combined with decreased 2-AG levels in GLAST-Dagla KO females^31^ could explain the greater reduction of appetite in the KOs compared with control mice, who show a significant increase in milk preference from proestrus to estrus phases. Moreover, levels of allopregnanolone, a neuroactive metabolite of progesterone, correspond to the levels of progesterone. Allopregnanolone is also associated with increases in food intake, preferences for energy-rich food, and obesity in humans and other mammals, and it stimulates food intake by activating GABA-A receptors in hypothalamus.^73^

In conclusion, we established a link between astrocytes, endocannabinoid production and the female sex hormones, which affects hedonic feeding. Mechanisms of how and when astrocytic *Dagla* affects sex hormone profiles have to be further elucidated. It is plausible that endocannabinoid production by astrocytes at least partly contributes to the greater susceptibility to overeating in females. Furthermore, this finding may be important for understanding the effects of exogenous cannabinoids on sex hormones.

## Supplementary Material

Supplemental data

## Data Availability

The datasets used and/or analyzed during this study are available from the corresponding author upon reasonable request.

## Abbreviations Used

2-AG: 2-arachidonoylglycerol
DAGLa: diacylglycerol lipase alpha
ECS: endocannabinoid system
BW: body weight
Cnr1: cannabinoid receptor 1
KO: knockout
FSH: follicle-stimulating hormone
LH: luteinizing hormone
ANOVA: analysis of variance
HPO: hypothalamic–pituitary–ovarian
