# Supplementary material for: Astrocytic Dagla Deletion Decreases Hedonic Feeding in Female Mice
Source: Cannabis Cannabinoid Res. 2024 Feb 12;9(1):74–88. doi: 10.1089/can.2023.0194 (PMC10874831; doi:10.1089/can.2023.0194)
Supplement: Supplemental data [file Suppl_Data.docx]

# Astrocytic *Dagla* deletion decreases hedonic feeding in female mice, Leidmaa et al

**Supplemental information**

**Materials and Methods**

## Animals

Male mice were injected with tamoxifen at 4-6 weeks and females at 8-10 weeks. Male mice were injected earlier because, in our initial experiment, mice from both control and KO groups injected with tamoxifen later than 8 weeks of age developed fat deposits in the testicular region. In female mice, the deletion of *Dagla* was induced in adulthood to avoid any programming effects by tamoxifen, as puberty has an organizational effect on how sex hormones affect feeding circuits in females (1,2). Experiments started 4 weeks after the tamoxifen injections. Mice that are heterozygous for Cre and homozygous for “floxed” (GLAST-Dagla KO) showed an astrocytic *Dagla* deletion after the induction with tamoxifen. Deletion of Dagla specifically from astrocytes and not from neurons or microglia was verified (3). The littermates not expressing the Cre gene that were homozygous for “floxed” *Dagla* (Dagla fl/fl) were used as controls. The "floxed" *Dagla* allele, the wild-type allele, the deleted *Dagla* allele, and the Cre locus were identified by polymerase chain reaction using appropriate primers (Dagla_fwd TAGCTTAGCCCCCATGTGAC, Dalga WT rev GAGATGGGTTCCACCTCCTT, Dagla fl/KO_rev CGCAGCCCAAAAGATACAAT, Cre1_fwd CATTTGGGCCAGCTAAACAT, Cre2_fwd GCATTTCTGGGGATTGCTTA, Cre1_rev CCCGGCAAAACAGGTAGTTA, Cre2_rev TGCATGATCTCCGGTATTGA). The presence of Cre under the GLAST promoter was validated by the GLAST-CreERT2-specific PCR (GlastCreERT2_ GAGGCACTTGGCTAGGCTCTGAGGA, GAGGAGATCCTGACCGATCAGTTGG, GGTGTACGGTCAGTAAATTGGACAT).

Animal care followed guidelines of the European Community’s Directive 86/609/EEC and the German Animal Protection Law regulating animal research. All experiments were approved by the North Rhine-Westphalia State Environment Agency (LANUV, Landesamt für Natur, Umwelt und Verbraucherschutz) and were performed in accordance with the relevant guidelines and regulations. The study was conducted in compliance with the ARRIVE guidelines.

## Food preference experiments

The milk solution was made of dairy cooking crème (Molkerei Weihenstephan, Germany) diluted in water to provide 1.64 kcal/g and a final composition of 5% fat, 1.3% carbohydrate, 1% protein (4). The milk preference test was also conducted after 24 h of fasting (food removed but water available). The intake of food, water and the test solutions were measured after 1, 3, 6, and 24 h. The milk preference test was stopped at the 6 h time-point since the milk spoiled at room temperature when kept for 24 h. Preference for palatable solutions was calculated over total liquid intake (solution of choice / (solution of choice + water)) x 100). We considered mice to have developed a preference when they showed more than a 75% preference for the solution of choice. Milk preference developed the fastest, after 1h of exposure, sucrose preference by 6 h and saccharine preference by 24 h, thus the respective time points are shown on graphs.

**References**

1. Massa MG, Correa SM. Sexes on the Brain: Sex as Multiple Biological Variables in the Neuronal Control of Feeding. Biochim Biophys Acta Mol Basis Dis. 2020 Oct 10;1866(10):165840.

2. Mauvais-Jarvis F. Sex differences in metabolic homeostasis, diabetes, and obesity. Biol Sex Differ. 2015 Dec 1;6(1):1–9.

3. Schuele LL, Glasmacher S, Gertsch J, Roggan MD, Transfeld JL, Bindila L, et al. Diacylglycerol lipase alpha in astrocytes is involved in maternal care and affective behaviors. Glia. 2021 Feb 1;69(2):377–91.

4. Leidmaa E, Gazea M, Patchev A V., Pissioti A, Christian Gassen N, Kimura M, et al. Blunted leptin sensitivity during hedonic overeating can be reinstated by activating galanin 2 receptors (Gal2R) in the lateral hypothalamus. Acta Physiologica. 2019;
